# Avulsion fracture of the coracoid process in a patient with chronic anterior shoulder instability treated with the Latarjet procedure: a case report

**DOI:** 10.1186/1752-1947-8-394

**Published:** 2014-11-29

**Authors:** Marco Michael Schneider, Maurice Balke, Paola Koenen, Bertil Bouillon, Marc Banerjee

**Affiliations:** 3 Department of Orthopaedic Surgery, Traumatology and Sports Medicine, Cologne-Merheim Medical Center, Witten/Herdecke University, Ostmerheimer Str. 200, D-51109 Cologne, Germany; 4 Department of Orthopaedic Surgery, Schulthess Clinic, Zurich, Switzerland; 5 Sportsclinic Cologne – Practice for Sportstraumatology, Cologne-Merheim Medical Center, Witten/Herdecke University, Ostmerheimer Str. 200, D-51109 Cologne, Germany

**Keywords:** Avulsion fracture, Coracoid process, Anterior shoulder instability, Latarjet, Recurrent shoulder dislocation

## Abstract

**Introduction:**

Shoulder dislocations can cause acute and chronic instabilities that need to be addressed in order to restore joint functioning. The transfer of the coracoid process has become a feasible surgical procedure in patients with shoulder instability. Several concomitant injuries after recurrent dislocations have been described.

**Case presentation:**

A 32-year-old German man presented to our department with a history of recurrent shoulder dislocations. He was diagnosed with an avulsion fracture of the coracoid process and dislocation of an osseous piece with attachment to the conjoined tendons during the surgical transfer of the coracoid process. Therefore, we performed an open Latarjet procedure and reattached the bony piece with the conjoined tendons to the glenoid rim. Three months after the operation the patient presented with a satisfying range of motion and without instabilities or pain. He was able to return to his job.

**Conclusions:**

Patients suffering from anterior shoulder dislocation might develop accompanying lesions after numerous dislocations that are not present upon first visit. Different techniques for the reconstruction of the glenoid rim and the restoration of shoulder joint stability have been described in the literature. We opted for a coracoid transfer and achieved an optimal reconstruction, as shown on the postoperative computed tomography scan. An avulsion fracture of the coracoid process with dislocation of the conjoined tendons can not only be found in patients suffering a direct trauma as pointed out in the literature, but also in patients with anterior shoulder instability with recurrent anterior shoulder dislocation.

## Level of evidence

Case Report, Level IV.

## Introduction

Recurrent anterior shoulder dislocations resulting in shoulder instability represent a surgical challenge due to ligamentous and bony defects of the glenoid rim and the humeral head. Various techniques have been described to address recurrent shoulder dislocations and subluxations. Arthroscopic and open procedures like the Bankart Repair or the Latarjet procedure are well known. The success of these surgical interventions is quantified by the rate of recurrence. The surgical transfer of the coracoid process has become a feasible option in patients suffering from chronic shoulder instability, especially in lesions accompanied by bony defects of the glenoid [1]. The goal of the Latarjet procedure is to stabilize the humeral head in the shoulder joint in two ways: firstly by enlarging the osseous joint surface and secondly by dynamic stabilization due to tension of the conjoined tendons [2, 3]. Prerequisites to perform the Latarjet procedure are an unimpaired coracoid process as well as intact conjoined tendons. A recurrent anterior shoulder dislocation with an accompanying avulsion fracture of the coracoid process has not been described in the literature.

## Case presentation

**Figure 1:**
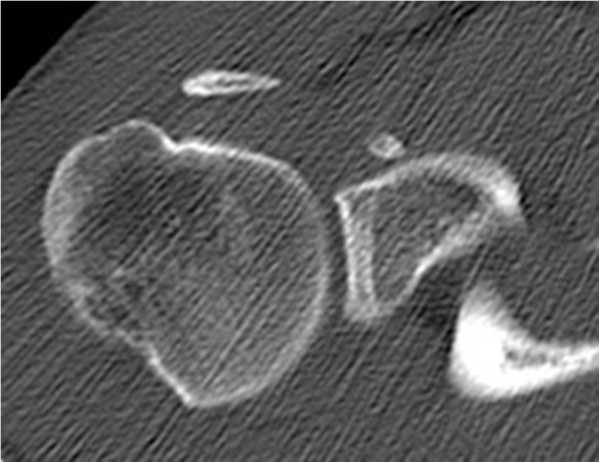
**Preoperative computed tomography scan showing the osseous piece.**

**Figure 2:**
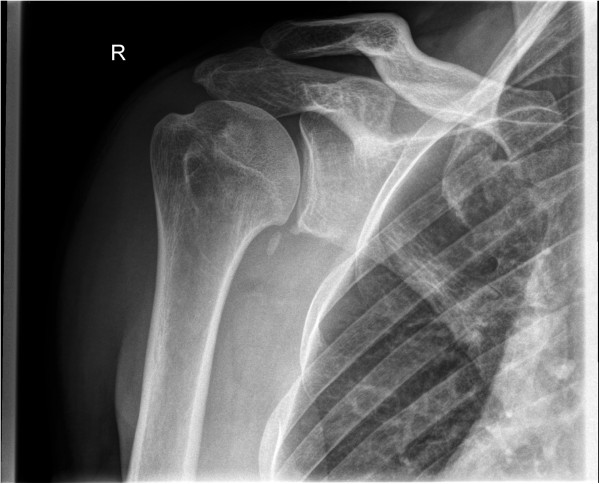
**Preoperative X-rays in anteroposterior projection.**

**Figure 3:**
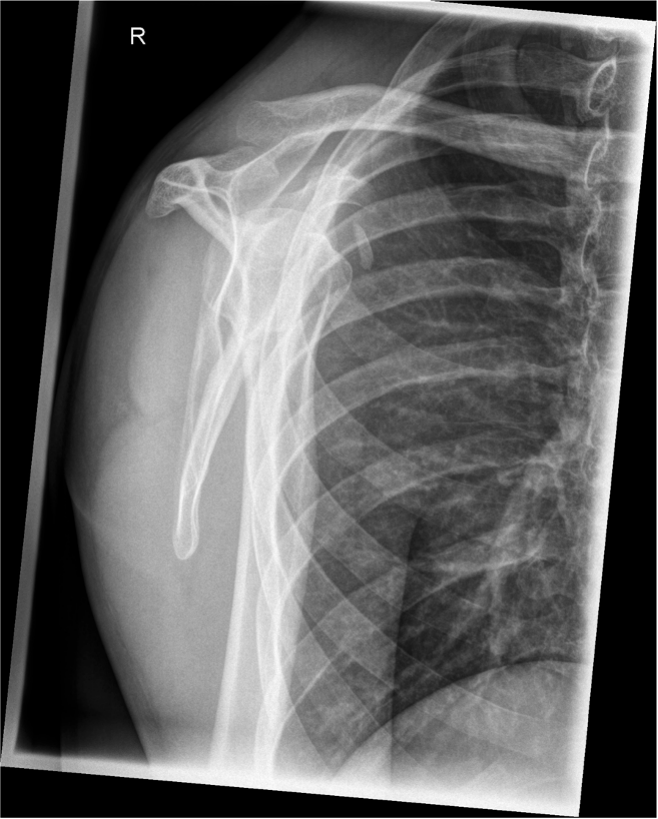
**Preoperative X-rays in lateral projection.**

A 32-year-old German man, employed as a railroad maintenance worker, presented to our department with chronic instability of his right shoulder. He reported that he suffered numerous anterior shoulder dislocations, which were reduced spontaneously every time. The initial work-related trauma occurred six years prior to the current presentation when he had lost consciousness due to exsiccosis and fell onto the railway line. His physical examination in our emergency room at that time showed a swollen right shoulder with pain-induced restriction in range of motion. His X-rays showed no signs of a luxation or fracture, so that we diagnosed a sprain of the acromioclavicular joint (Rockwood classification I). Since he lost consciousness he was not able to tell whether his shoulder might have been dislocated. A magnetic resonance imaging (MRI) scan taken six months later showed a subdeltoid bursitis without evidence of a rotator cuff tear or osseous pathology. Upon his visit six months after the injury his right shoulder an Anterior Apprehension Test revealed an anterior shoulder instability, while the range of motion, the rotator cuff, sensibility and circulation seemed intact on clinical examination. A conservative treatment with physiotherapy was initiated. Six years later he presented to our department again complaining of chronic anterior shoulder instability with spontaneous reduction without a second trauma. His Apprehension and Relocation tests were positive for the mentioned anterior shoulder instability while a posterior instability could be excluded. A further MRI scan displayed a Hill-Sachs lesion of the humeral head and a lesion of the anterior labrum with a possible osseous involvement. After completing standard X-rays as well as a computed tomography (CT) scan that revealed a glenoid rim defect in addition to the above-mentioned defects, we advised surgery and he was scheduled for an open Latarjet procedure (Figures 1, 2 and 3).

**Figure 4:**
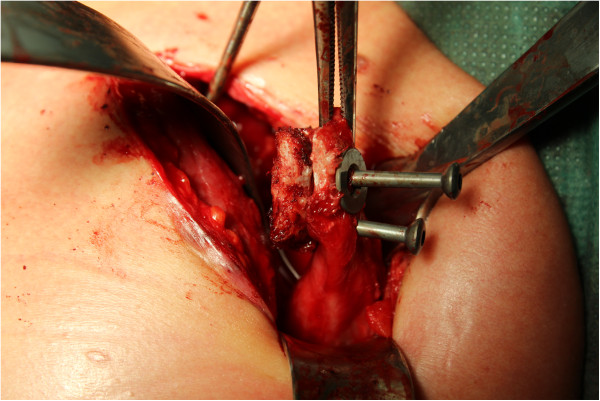
**Linking of the osseous piece to the detached coracoid process.**

**Figure 5:**
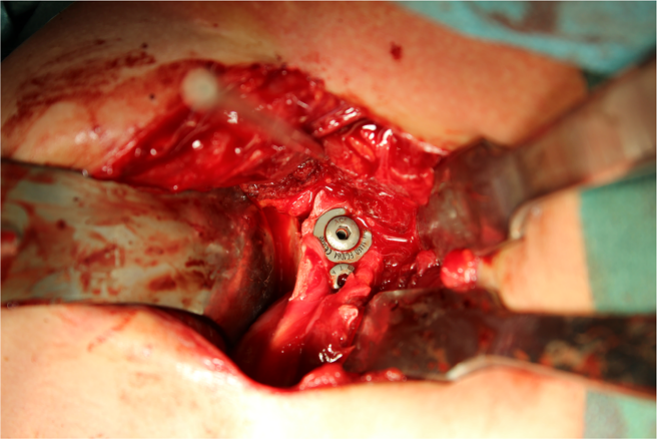
**Fixation of the coracoid process with the attached osseous piece to the glenoid rim.**

The examination under anesthesia confirmed the anterior shoulder instability. The operation was performed in the beach chair position. After sterile cleaning and draping we used the deltopectoral approach. After preparation and sectioning of the clavipectoral fascia we displayed the coracoid bone. At that stage we noticed that the conjoined tendons were not attached to the coracoid process. The tendon of the pectoralis minor, the short head of the biceps brachii and the coracobrachialis muscle were proximally ossified about 2cm lateral of the coracoid process. We concluded that he suffered an avulsion fracture of the coracoid process. The coracoid process was then transferred in an open Latarjet procedure, with re-fixation of the avulsion fracture with attached conjoined tendons to the glenoid rim. We used two 4mm screws (length 26mm and 32mm) for re-fixation of the coracoid process and the bone fragment (Figures 4 and 5).

**Figure 6:**
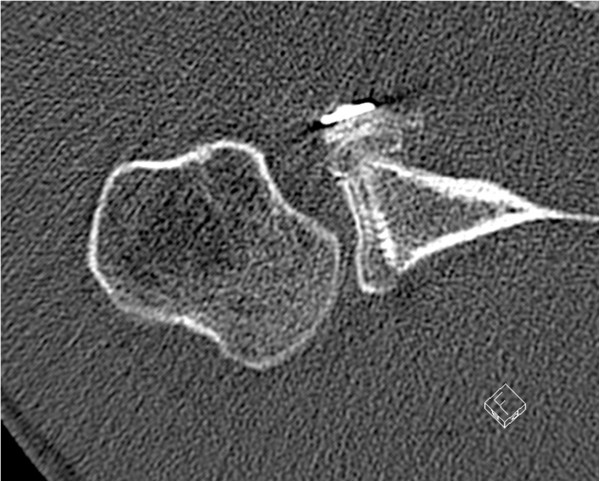
**Postoperative computed tomography scan for determination of the position of the coracoid process with attached osseous piece taken immediately after the intervention.**

His immediate postoperative CT scan showed an optimal position of the coracoid process as well as the osseous piece attached to the conjoined tendons (Figure 6). After the procedure, his shoulder was immobilized in an arm sling for two weeks. Passive physiotherapy (90° anteversion, 90° abduction, 30° outer rotation) was recommended for six weeks. After completing this course of physiotherapy he was allowed to move his shoulder actively in every direction. Contact sports were allowed approximately four months after verification of proper bone remodeling.

**Figure 7:**
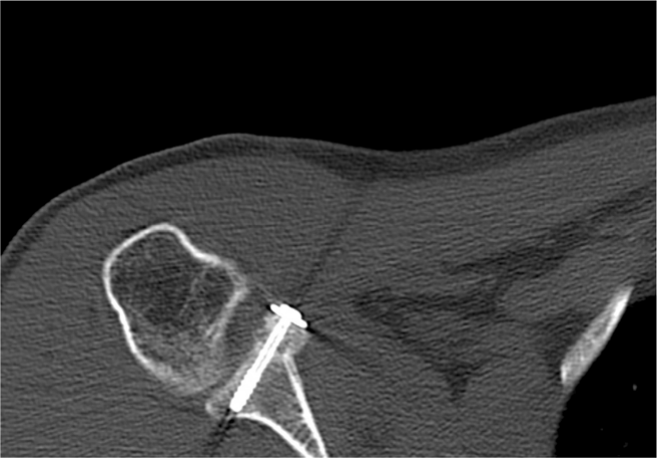
**Three-month follow-up computed tomography scan with timely ossification of the attached bone piece.**

On his follow-up visit three months after the intervention he presented with a satisfying range of motion and without pain (140° anteversion, 120° abduction, 40° external rotation). All instability tests were negative. A repeated CT scan showed a timely ossification of the fixed bony piece (Figure 7). Therefore, full weight bearing was allowed and he was motivated to return to his job.

## Discussion

Anterior glenohumeral dislocations represent the most common of all dislocations. The primary trauma may lead to chronic shoulder instability. Bone defects of the glenoid rim are a frequent result of recurrent dislocations in the shoulder joint [4–6]. A reconstruction of the osseous lesions is recommended in patients with relevant bone loss [7–9]. Another impact of continuing shoulder dislocations might be a fracture of the coracoid process, which usually occurs in the company of acromioclavicular joint dislocations. However, Eyres *et al.* as well as Kalicke *et al.* have described a fracture of the coracoid process as a rare complication in patients with anterior shoulder dislocation [10, 11]. In our case, the recurrent anterior glenohumeral dislocations led to a chronic instability and to an avulsion fracture of the coracoid process with the attached conjoined tendons. An isolated demolition of the coracoid process is unique and mostly treated conservatively [10, 12, 13]. A dislocation of an osseous piece attached to the conjoined tendons as concomitant injury after recurrent anterior shoulder dislocations has not been previously described in the literature to the best of our knowledge. Different studies have shown that recurrent shoulder dislocations can lead to various accompanying lesions that develop over time and are not present after primary shoulder dislocation [14]. Upon our patient’s first visit we suspected an acromioclavicular joint sprain without further bony or ligamentous lesions. Almost seven years and numerous luxations with spontaneous repositions later, he presented to our department again, this time with the abovementioned concomitant injuries such as a glenoid rim defect, a Hill-Sachs lesion and an avulsion fracture of the coracoid process. The exact date and mechanism of the avulsion fracture is not determinable.

The Latarjet procedure has been described as feasible option for treating shoulder instabilities even after failed operative repair [15]. In addition to the osseous enlargement of the glenoid rim, the sling effect of the conjoined tendons is considered an essential part for regaining stability of the shoulder joint [2, 3]. Therefore we decided to perform an open Latarjet procedure in our surgical intervention. Intraoperatively, we identified the osseous piece with attachment of the conjoined tendons as quite big, so a sole re-fixation of the bone fragment probably would have induced enough stability in the shoulder joint. It can be debated which operative technique would have been most suitable: a sole re-fixation of the avulsed osseous piece with the adjacent conjoint tendons, a glenoid reconstruction with an iliac crest bone graft or the utilization of an open coracoid process transfer.

Retrospectively an avulsed piece of bone can be detected in the preoperative CT and probably even in the preoperative X-rays, taken six years after the initial trauma (Figures 6 and 1). However, it still remains difficult to assign the osseous fragment to the coracoid process. Since the literature did not describe the combination of anterior shoulder instability, a bony glenoid defect and an avulsion fracture of the coracoid process with the adjacent conjoined tendons we did not consider this rare combination; he presented without a loss of strength or impairment of shoulder movement upon his visits in our department.

## Conclusions

Recurrent anterior shoulder dislocations may cause several complications over time that are not present upon a patient’s first visit. Depending on the extent of the bony defect of the glenoid rim a surgery (Bankart Repair, iliac crest bone graft or Latarjet Procedure) is recommended. We would like to point out that an avulsion fracture of the coracoid process might be another possible concomitant injury, although the patient might present as asymptomatic. A preoperative CT scan is performed in most cases and might likely give an indication towards this type of injury, if one knows that this accompanying lesion can occur (Figure 1). Furthermore, it might be important to identify the presence of such an injury preoperatively in case of an arthroscopic Latarjet procedure, as it may be impossible to gain the same results afterwards.

## Consent

Written informed consent was obtained from the patient for publication of this case report and any accompanying images. A copy of the written consent is available for review by the Editor-in-Chief of this journal.

## Abbreviations

CT: Computed Tomography
MRI: Magnetic Resonance Imaging.
